# Invasion Risk of Established and Horizon Non-Native Ants in the Mediterranean: A Screening for Italy

**DOI:** 10.3390/insects15110875

**Published:** 2024-11-08

**Authors:** Enrico Schifani, Daniela Giannetto, Lorenzo Vilizzi

**Affiliations:** 1 Life Sciences and Environmental Sustainability, Department of Chemistry, University of Parma, 43124 Parma, Italy; 2 Faculty of Science, Department of Biology, University of Mugla Sitki Kocman, Mugla 48000, Türkiye; 3 Faculty of Biology and Environmental Protection, Department of Ecology and Vertebrate Zoology, University of Lodz, 90-237 Lodz, Poland; lorenzo.vilizzi@gmail.com; 4 Department of Biological Sciences, College of Science, Research Center for the Natural and Applied Sciences, The Graduate School, University of Santo Tomas, Manila 1015, Philippines

**Keywords:** climate change, decision support tools, red imported fire ant, Argentine ant, electric ant, Asian needle ant, terrestrial species invasiveness screening kit (TAS-ISK)

## Abstract

Over five hundred non-native ant species have spread worldwide, including many that have severe effects on biodiversity, are serious economic pests, or threaten human health and agriculture. The number of species in the Mediterranean is steadily increasing, with Italy being a prominent example. We provide risk screenings for non-native ant species in Italy using a Terrestrial Species Invasiveness Screening Kit using current climate conditions and future predictions. The screened species consist of 15 established and 12 horizon taxa. The results highlight the threat posed by *Linepithema humile* and *Solenopsis invicta*, followed by *Wasmannia auropunctata*, *Brachyponera chinensis*, and *Solenopsis geminata*. The threat posed by other tropical invaders such as *Anoplolepis gracilipes* and *Pheidole megacephala* depends on climate change scenarios. The Palearctic non-native *Lasius neglectus* and *Tetramorium immigrans* species are recognized as intermediate threats, while most screened species are far less threatening. The biology and ecology of most non-native ant species remain scarcely documented. Among the established species, *B. chinensis*, *L. humile*, and *S. invicta* deserve the most attention, while *W. auropunctata* is rapidly spreading in neighboring countries. Detection is still often accidental and late compared to establishment. Most species first establish around urban areas, making citizen science a promising tool for biosurveillance.

## Simple Summary

Invasive ant species are increasingly proving dangerous to native biodiversity and ecosystems, agriculture, other economic activities, and human health. Like many Mediterranean countries, Italy is witnessing a steady increase in non-native ant species of different origins and with different biological characteristics. Climate change is further posed to alter the region’s suitability for non-native ants; therefore, assessing their invasion potential is a crucial step in developing management strategies. We provide risk screenings for 15 non-native ant species already established in Italy and 12 that may be established in the future using a Terrestrial Species Invasiveness Screening Kit. The results indicate the Argentine ant, *Linepithema humile*, and the red imported fire ant, *Solenopsis invicta*, to be the most threatening species, followed by the electric ant, *Wasmannia auropunctata*; the Asian needle ant, *Brachyponera chinensis*; and the tropical fire ant, *Solenopsis geminata*. The harmfulness of other tropical species largely varies based on climatic predictions, while most species are far less dangerous. However, the impact of many ants is still undocumented, and the future role of climate change in their invasiveness is unclear. The detection of newly established species is often late and accidental, but public engagement could be crucial as most species first establish near cities.

## 1. Introduction

More than five hundred ant species have been introduced outside their native range by human activities, almost always unintentionally. Some of these species are ranked among the world’s most damaging invasive species due to their environmental and economic impacts [1,2,3,4] and nineteen are listed in the Global Invasive Species Database [5]. Among invasive ants, some act as pests in agricultural and urban environments and threaten human health in a few cases [1,2,3]. A few successful species can cause severe disruption to the invaded ecosystems, displacing native ants and affecting other invertebrates, plants, and vertebrates through direct interactions and cascading effects [1,4]. At the same time, most non-native ants are restricted to heavily disturbed habitats, where they sometimes depend on special microclimatic conditions (e.g., heated buildings, greenhouses, irrigated gardens) and are considered to have little or no impact [1,4,6]. It is therefore crucial to distinguish between different non-native ant species and their invasion potential to plan management and control actions.

The Mediterranean region stands as a key terrestrial biodiversity hotspot, hosting native and rare ant species [7,8]. However, a steady and threatening increase in the number of non-native ant species has occurred over the last few decades [9,10,11,12]. Data from cargo hotspots indicate that an even larger number of non-native ant species may arrive [13]. Ants are among the most difficult invasive species to control because, given their small size, they are hard to detect and can easily spread without being noticed [14,15]. However, no invasion risk assessment has so far been conducted for non-native ants in the Mediterranean Region. Some introduced species have attracted significant attention from both scientists and media outlets. This is the case with the Argentine ant, *Linepithema humile*, first recorded in Portugal at the end of the 19th century [16], and the relatively recent arrivals of the red imported fire ant, *Solenopsis invicta*, in Italy [11,17] and the electric ant, *Wasmannia auropunctata*, in Cyprus, France, and Spain [12,18,19]. Conversely, other species known to cause serious impacts in other areas of the world have so far seemed to pose little threat to Mediterranean habitats, even following their occasional introduction (e.g., the African big-headed ant, *Pheidole megacephala* [20]). In Europe, while most established ant species are limited to urban or agricultural habitats, many of them can establish outdoors in Euro-Mediterranean countries [9,21,22]. Climate change represents a further source of uncertainty for the establishment potential of non-native ants in Europe, with non-anecdotal predictions currently available for only a handful of species [11,23,24,25]. 

Italy hosts an increasing number of non-native ant species [9,26]. Recent discoveries include threatening species, such as the red imported fire ant and the Asian needle ant, *Brachyponera chinensis*, as well as *Hypoponera ergatandria* and *Nylanderia vividula*, bring the total number of established ants to at least 15 [10,11,27,28]. Although the ecological and economic threats posed by some of these non-native ant species have been well documented [1,11], there is a notable lack of information about the potential invasiveness of most of them. This gap makes effective management and control efforts challenging [2,3]. This study aims to address this deficiency by providing risk screenings for established and potentially invasive non-native ants in Italy. The objective is to provide a thorough evaluation of the risk potential for each species by applying a recently released decision support tool and considering both present and future climate predictions. This comprehensive method will be useful to promote the effective management and control of these species in Italy and will represent a valuable background for the evaluation of comparable risk screenings in other regions threatened by non-native ant species.

## 2. Materials and Methods

To identify potentially invasive non-native ants in Italy (the risk assessment area), a risk screening was conducted involving 27 species (Table 1). These included 15 species established in Italy (*Brachyponera chinensis, Hypoponera ergatandria, Hypoopnera punctatissima, Lasius neglectus, Linepithema humile, Monomorium pharaonis, Nylanderia jaegerskioeldi, Nylanderia vividula, Paratrechina longicornis, Pheidole indica, Solenopsis invicta, Strumigenys membranifera, Tetramorium bicarinatum, Tetramorium immigrans,* and *Tetramorium lanuginosum*) plus 12 horizon species chosen for their invasive potential and presence in the Mediterranean or in the whole of Europe (*Anoplolepis gracilipes, Brachymyrmex patagonicus, Cardiocondyla obscurior, Monomorium carbonarium, Monomorium floricola, Pheidole megacephala, Plagiolepis alluaudi, Solenopsis geminata, Tapinoma melanocephalum, Technomyrmex pallipes, Trichomyrmex destructor,* and *Wasmannia auropunctata*) [9,10,11,12,13,18,19,21,22,26,27,28,29,30]. The black imported fire ant, *Solenopsis richteri*, a species of concern according to the European Union, was not screened as it was deemed to be very similar to the red imported fire ant, *Solenopsis invicta*, while the remaining invasive species of Union concern according to the latest list (Commission Implementing Regulation (EU) 2022/1203 of 12 July 2022, amending Implementing Regulation (EU) 2016/1141 to update the list of invasive non-native species of Union concern) were all included: the aforementioned *Solenopsis invicta* and *Wasmannia auropunctata* and the tropical fire ant, *Solenopsis geminata* [5]. For the genera *Nylanderia* and *Technomyrmex*, both including several introduced species worldwide whose proper recognition has suffered from taxonomic difficulties, only the representatives that have a wider presence in the region were screened.

Risk screening was undertaken using the Terrestrial Animal Species Invasiveness Screening Kit (TAS-ISK v2.4 [32]). This multilingual, taxon-generic decision support tool complies with the ‘minimum standards’ for screening non-native species under EC Regulation No. 1143/2014 on the prevention and management of the introduction and spread of invasive species [33]. The TAS-ISK consists of 55 questions of which 49 comprise the Basic Risk Assessment (BRA) and six the Climate Change Assessment (CCA). The latter component requires the assessor to predict how future predicted climatic conditions are likely to affect the BRA concerning risks of introduction, establishment, dispersal, and impact. All screenings were carried out by the first author. 

The screening process followed the standard protocol by Vilizzi et al. [31], with the assessor providing a response, a confidence level, and a justification for each question [34]. Upon completion of a species’ screening, the BRA and BRA+CCA scores are computed. In both cases, a score < 1 indicates a ‘low risk’ of the species being or becoming invasive in the risk assessment area, whereas a score ≥ 1 indicates a ‘medium risk’ or a ‘high risk’. Distinction between medium-risk and high-risk species is made by computing a calibrated threshold through Receiver Operating Characteristic (ROC) curve analysis [31,35]. A measure of the accuracy of the calibration analysis is the area under the curve (AUC), whose values are interpreted as follows: 0.7 ≤ AUC < 0.8 = acceptable discriminatory power, 0.8 ≤ AUC < 0.9 = excellent, 0.9 ≤ AUC = outstanding [36]. An additional ad hoc threshold was also defined to distinguish within species classified as high risk those carrying a ‘very high risk’ of invasiveness (as per [37]). Following the identification of the threshold, an evaluation of the risk rankings to identify false/true negative/positive outcomes was not applied to the medium-risk species because their evaluation in a follow-up risk assessment depends on both management priorities and the availability of financial resources [31]. 

The a priori categorization of species to implement ROC curve analysis followed Vilizzi et al. [31]. Fitting of the ROC curve was performed with pROC [38] for R x64 v4.3.2 [39]. Permutational ANOVA following normalization of the data was used to test for differences in the confidence factor (CF: see [31]) between the BRA and BRA + CCA using a Bray–Curtis dissimilarity measure, 9999 unrestricted permutations of the raw data, and with statistical effects evaluated at α = 0.05.

## 3. Results

The ROC curve analysis resulted in a threshold of 16.5 and an AUC of 0.8294 (0.6602–0.9986 95% CI); hence, it showed excellent discriminatory power. The threshold was therefore used for the calibration of the BRA and BRA+CCA scores to distinguish between medium- and high-risk species under current and predicted climate conditions, respectively (Table 2). 

**Figure 1:**
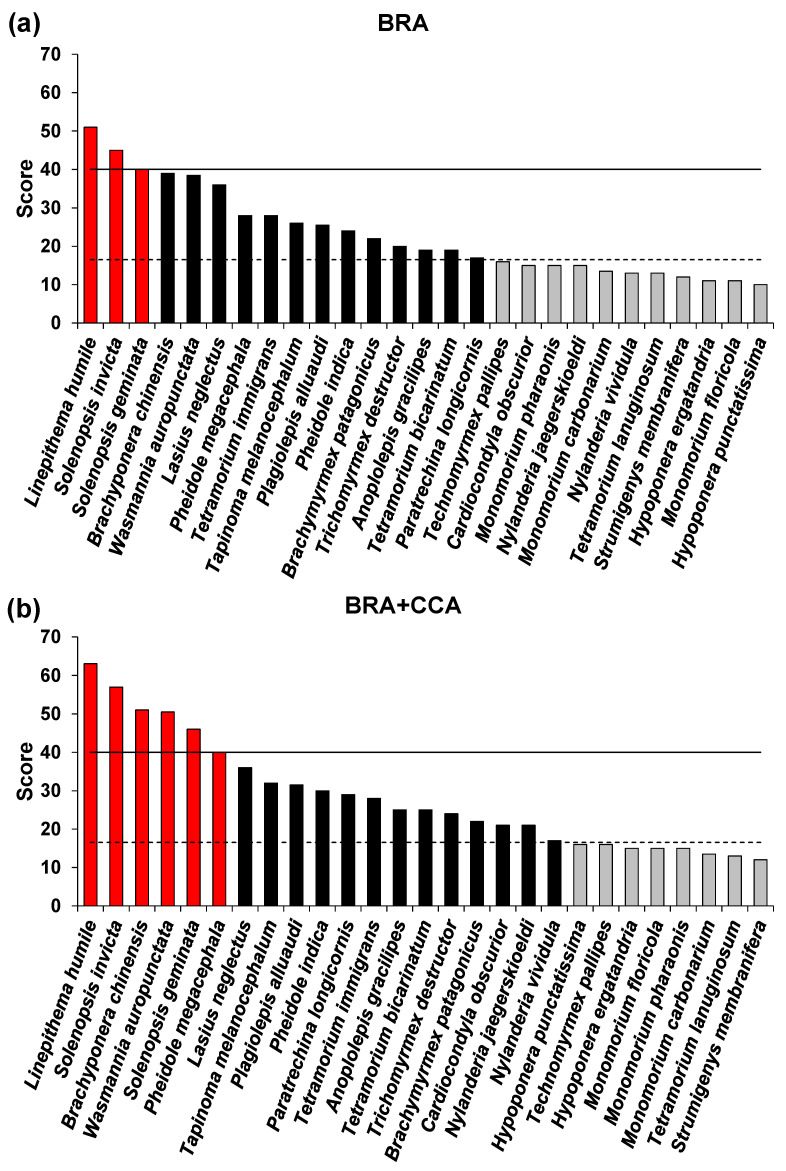
Risk outcome scores for the non-native ants screened with the Terrestrial Animal Species Invasiveness Screening Kit (TAS-ISK) in Italy. (**a**) Basic Risk Assessment (BRA) scores; (**b**) BRA + Climate Change Assessment (BRA+CCA) scores. Red bars = very-high-risk species. Black bars = high-risk species. Gray bars = medium-risk species. Solid line = very-high-risk (VH) threshold. Hatched line = high-risk (H) threshold. Thresholds as per Table 2.

Based on the BRA scores (Table 2, Figure 1a), 3 (11.1%) species were ranked as very high risk, 13 (48.2%) as high risk, and 11 (40.7%) as medium risk. Of the 17 species categorized a priori as invasive, 3 were ranked as very high risk (Argentine ant, *Linepithema humile*; tropical fire ant, *Solenopsis geminata*; red imported fire ant, *Solenopsis invicta*) and 13 as high risk (true positives: yellow crazy ant, *Anoplolepis gracilipes*; dark rover ant, *Brachymyrmex patagonicus*; Asian needle ant, *Brachyponera chinensis*; invasive garden ant, *Lasius neglectus*; longhorn crazy ant, *Paratrechina longicornis*; Indian big-headed ant, *Pheidole indica*; African big-headed ant, *Pheidole megacephala*; ghost ant, *Tapinoma melanocephalum*; tramp ant, *Tetramorium bicarinatum*; destroyer ant, *Trichomyrmex destructor;* and electric ant, *Wasmannia auropunctata*). Of the ten species categorized a priori as non-invasive, two were ranked as high risk (false positive: little yellow ant, *Plagiolepis alluaudi*, and pavement ant, *Tetramorium immigrans*). Of the eleven medium-risk species, eight were a priori non-invasive (*Hypoponera ergatandria*; Roger’s ant, *Hypoponera punctatissima*; *Monomorium carbonarium*; *Nylanderia jaegerskioeldi*; *Nylanderia vividula*; membraniferous dacetine ant, *Strumigenys membranifera*; white-footed ant, *Technomyrmex pallipes*; wooly ant, *Tetramorium lanuginosum*) and three were invasive (*Cardiocondyla obscurior*, bicolored trailing ant, *Monomorium floricola*; pharaoh ant, *Monomorium pharaonis*). 

Based on the BRA+CCA scores (Table 2, Figure 1b), 19 (70.4%) species were ranked as very high or high risk and 8 (29.6%) we ranked as medium risk. Of the a priori invasive species, 15 were ranked as high or very high risk (same species as for the BRA plus *Cardiocondyla obscurior*), and of the a priori non-invasive species 4 were ranked as high risk (same species as per BRA plus *Nylanderia jaegerskioeldi* and *Nylanderia vividula*). Of the eight medium-risk species, six were a priori non-invasive (*Hypoponera ergatandria*, *Hypoponera punctatissima*, *Monomorium carbonarium*, *Strumigenys membranifera*, *Technomyrmex pallipes*, *Tetramorium lanuginosum*) and two were invasive (*Monomorium floricola* and *Monomorium pharaonis*). 

Based on an ad hoc threshold ≥40, *Linepithema humile*, *Solenopsis invicta*, and *Solenopsis geminata* were ranked as very high risk for both the BRA and BRA+CCA, and an additional three species were ranked for the BRA+CCA only (i.e., *Brachyponera chinensis*, *Pheidole megacephala*, and *Wasmannia auropunctata*) (Table 2, Figure 1). The number of species ranked as high (and very high) risk increased from 16 (59.3%) under the BRA to 19 (70.4%) under the BRA+CCA. The CCA resulted in an increase in the BRA score (cf. BRA+CCA score) for 16 (70.4%) species and in no change for 8 (29.6%) (Table 2). 

The mean CF_Total_ was 0.741 ± 0.012 SE, the mean CF_BRA_ was 0.779 ± 0.011 SE, and the mean CF_CCA_ was 0.427 ± 0.044 SE, hence indicating lower confidence for the CCA (Table 2). The mean CF_BRA_ was higher than mean CF_CCA_ (F^#^_1,52_ = 60.35, *p*^#^ < 0.001; # = permutational value).

## 4. Discussion

This study represents the first risk screening focusing on non-native ant species in a Mediterranean country and the second in a European country after the one by Báthori et al. [40]. The results fill a knowledge gap by providing a comprehensive evaluation of the risk posed by non-native ant species in a representative country of the Mediterranean region and provide a valuable framework for future risk assessment. This study also contributes to increasing the knowledge of the potential hazards posed by the screened species, underlining the need for focused monitoring and management measures considering the increasingly complex ecological interactions triggered by climate change. 

*Linepithema humile* and *Solenopsis invicta* were the species associated with the highest risk scores under both current and predicted climate conditions. There is an overwhelming body of literature describing the environmental impact of both species, which are regarded among the worst invasives globally [4,24,41,42]. *Linepithema humile* is the only invasive species that has already proved to be highly capable of invading and deteriorating Mediterranean ecosystems, with an extraordinary supercolonial organization ranging through southwestern Europe [42]. It is mainly associated with coastal and highly disturbed habitats in Italy, where it was first detected in the early 20th century [16]. On the other hand, *Solenopsis invicta* has only recently been discovered in Italy, the first European or Mediterranean country to witness its establishment, which may have occurred since at least 2015 [10,11]. While both species are considered serious ecological threats and can also harm agricultural activities, *Solenopsis invicta* is also a threat to electric infrastructures and human health [41]. Both species are expected to significantly expand their suitable range in Europe due to climate change [10,23]. 

A high risk was also associated with *Wasmannia auropunctata*, *Solenopsis geminate*, and *Brachyponera chinensis*, which are all ecologically damaging and regarded as health threats due to their stinging abilities [3,43,44]. *Brachyponera chinensis* has been found in two far-apart Italian localities very recently, and is otherwise unknown elsewhere in Europe, while its invasive abilities were mostly studied in North America [10,28]. On the other hand, *Wasmannia auropunctata*, traditionally considered a tropical species, has been detected in three Euro-Mediterranean countries very recently, establishing viable and growing populations [12,18,19,45]. Finally, while past introductions of *Solenopsis geminata* in the region did not result in established outdoor populations or were based on misidentifications, a potential invasion of the species is still considered high risk due to its invasive abilities, environmental impact, and powerful sting, and it has been included in the list of species of Union concern by the EU alongside *Solenopsis invicta*, *Solenopsis richteri,* and *Wasmannia auropunctata* [22,46]. 

When climate change scenarios are considered, there is an increase in the risk level associated with several species, most evident in *Pheidole megacephala, Paratrechina longicornis, Solenopsis invicta,* and *Wasmannia auropunctata*. Among the species with increased risk levels, *Solenopsis geminata*, *Pheidole megacephala*, and *Anoplolepis gracilipes* have a serious invasive record in the tropics but never had significant success in the Mediterranean in past introductions. None of them seems to be currently established in Italy (*Pheidole megacephala* only temporarily established at Malpensa airport, Milan (see [13])). While their suitability models suggest a future increase in Europe, this is not necessarily decisive enough or the evidence is not conclusive [47,48,49]. In general, most of the species assessed are native to warm tropical climates and are predicted to increase their invasion risk with climate change, but predictions are still often anecdotal (low confidence) and these results must be treated accordingly.

Two Palearctic species supposedly native to Anatolia and Caucasus or Central Asia, namely *Lasius neglectus* and *Tetramorium immigrans*, have somewhat intermediate invasion risk scores: the two have been successfully colonizing urban and disturbed habitats across Europe and have locally been considered pests, with *Tetramorium immigrans* being a cryptic invader of recent recognition and unknown introduction time that is particularly widespread [50,51,52,53]. Most of the species have lower scores, as none of them are expected to pose a significant threat to either the environment or human activities, and most are likely to be confined within buildings or near urban areas [9]. However, information on their ecological role is extremely scarce in most cases—including for those species that have already established in Italy or the Mediterranean region. 

The number of non-native ants in Italy rapidly passed from six at the beginning of the 21st century [9] to the current number of at least fifteen [10,11,27,28]. Most species discovered during the last few years have been spotted near urban areas, and most were first detected by non-professional researchers, pest controllers, or curious citizens before being identified by specialists [9,10,11,13,17,27,28,29]. This pattern is overall similar across different European countries, with stinging species attracting significant attention [9,10,11,12,17,19,28]. While dangerous lag is often observed between first establishment and detection, given non-native ants’ preference for anthropogenic habitats, citizen science and public engagement could play a key role in aiding biosurveillance efforts and achieving earlier detection of threats [17,54]. However, the increasing number of either established in Italy and in Mediterranean Europe, in general, requires us to establish priorities and distinguish between species posing different threats. Hence, the use of TAS-ISK and similar decision tools to evaluate the risks posed by non-native species could be useful to monitor the risk posed by incoming species and implement control and management strategies. 

## 5. Conclusions

The present results clearly suggest that some species, like the already established *Linepithema humile* and *Solenopsis invicta*, are those posing the highest risk under both current and predicted climate conditions. The concurrence of their well-known capacities to negatively impact biodiversity with all associated costs, together with their high potential to rapidly expand their range of invasion because of climate change, suggest that the urgent implementation of monitoring and management measures should become a priority. The results of this study also highlight the importance of integrating decision support tools like TAS-ISK to evaluate the levels of threat posed by different species in order to promote a more strategic allocation of management efforts. Also, the risks associated with some species that are traditionally not successful in the Mediterranean appear to be worsened under predicted climate change scenarios. 

Public outreach and citizen science programs are promising considering the increasing number of non-native ant species that are being reported from Italy and which are frequently detected for the first time by non-specialists, often with a significant delay from their establishment. While their correct identification normally requires highly trained taxonomists, increasing public participation and awareness can improve early detection and monitoring initiatives. In addition to official surveillance efforts, programs that would promote the reporting of new non-native ant species occurrences and assist and increase awareness about the potential consequences of these species can be useful to enhance biosurveillance measures in general. Effective monitoring and management measures are important considering the growing number of non-native species and the potential risks they pose. More in-depth research about the ecological effects of less-studied species, especially those with lower risk scores, is also encouraged.

## Figures and Tables

**Table 1:** Non-native ant species screened with a Terrestrial Animal Species Invasiveness Screening Kit (TAS-ISK) for their invasion risk in Italy. The a priori categorization follows the four-step protocol of Vilizzi et al. [31]: (1) FishBase (www.fishbase.org (accessed on 13 September 2024)); (2) Global Invasive Species Database (GISD: www.iucngisd.org (accessed on 13 September 2024)); (3) European Alien Species Information Network (EASIN: https://easin.jrc.ec.europa.eu/easin (accessed on 13 September 2024)); (4) Google Scholar literature search. N = no impact/threat; Y = impact/threat; ‘–’ = absent; n.a. = not applicable. Species considered established in Italy are marked in bold.

		A priori Categorization				
Species Name	Common Name	GBIF	GISD	EASIN	Google Scholar	Outcome
*Anoplolepis gracilipes*	yellow crazy ant	Y	Y	–	n.a.	Invasive
*Brachymyrmex patagonicus*	dark rover ant	N	–	N	Y	Invasive
*Brachyponera chinensis*	Asian needle ant	N	–	N	Y	Invasive
*Cardiocondyla obscurior*	–	Y	–	N	n.a.	Invasive
*Hypoponera ergatandria*	–	N	–	N	N	Non-invasive
*Hypoponera punctatissima*	Roger’s ant	N	–	N	N	Non-invasive
*Lasius neglectus*	invasive garden ant	N	Y	Y	n.a.	Invasive
*Linepithema humile*	Argentine ant	N	Y	Y	n.a.	Invasive
*Monomorium carbonarium*	–	N	–	–	N	Non-invasive
*Monomorium floricola*	bicolored trailing ant	Y	Y	N	n.a.	Invasive
*Monomorium pharaonis*	pharaoh ant	N	Y	Y	n.a.	Invasive
*Nylanderia jaegerskioeldi*	–	N	–	–	N	Non-invasive
*Nylanderia vividula*	–	N	–	–	N	Non-invasive
*Paratrechina longicornis*	longhorn crazy ant	N	Y	Y	n.a.	Invasive
*Pheidole indica*	Indian big-headed ant	N	–	–	Y	Invasive
*Pheidole megacephala*	African big-headed ant	N	Y	Y	n.a.	Invasive
*Plagiolepis alluaudi*	little yellow ant	N	–	N	N	Non-invasive
*Solenopsis geminata*	tropical fire ant	Y	Y	Y	n.a.	Invasive
*Solenopsis invicta*	red imported fire ant	Y	Y	Y	n.a.	Invasive
*Strumigenys membranifera*	membraniferous dacetine ant	N	–	–	N	Non-invasive
*Tapinoma melanocephalum*	ghost ant	Y	Y	Y	n.a.	Invasive
*Technomyrmex pallipes*	white-footed ant	-	–	N	N	Non-invasive
*Tetramorium bicarinatum*	tramp ant	N	–	Y	n.a.	Invasive
*Tetramorium immigrans*	pavement ant	N	–	–	N	Non-invasive
*Tetramorium lanuginosum*	wooly ant	N	–	N	N	Non-invasive
*Trichomyrmex destructor*	destroyer ant	Y	–	–	n.a.	Invasive
*Wasmannia auropunctata*	electric ant	Y	Y	Y	n.a.	Invasive

**Table 2:** Risk outcomes for the non-native ant species screened with the TAS-ISK for Italy. For each species, the following information is provided: a priori categorization of invasiveness (N = non-invasive; Y = invasive: see Table 1); Basic Risk Assessment (BRA) and BRA + Climate Change Assessment (BRA + CCA) scores with corresponding risk ranks based on a calibrated threshold of 16.5 (M = medium; H = high; VH = very high, based on an ad hoc threshold ≥ 40. See text for details); classification (Class: FP = false positive; TP = true positive; ‘–’ = not implemented as medium risk; n.a. = not applicable. See text for details); CCA as the difference between BRA + CCA and BRA scores; and confidence factor (CF). Risk outcomes for the BRA scores (within the interval): M [1, 16.5[, H ]16.5, 40[, VH [40, 72]. Risk outcomes for the BRA + CCA scores: M [1, 16.5[, H ]16.5, 40[, VH [40, 82]. Note the reverse bracket notation indicating an open interval.

		BRA			BRA + CCA				CF		
Species Name	A priori	Score	Rank	Class	Score	Rank	Class	CCA	Total	BRA	CCA
*Anoplolepis gracilipes*	Y	19.0	H	TP	25.0	H	TP	6	0.65	0.70	0.25
*Brachymyrmex patagonicus*	Y	22.0	H	TP	22.0	H	TP	0	0.69	0.74	0.25
*Brachyponera chinensis*	Y	39.0	H	TP	51.0	VH	TP	12	0.74	0.73	0.79
*Cardiocondyla obscurior*	Y	15.0	M	–	21.0	H	TP	6	0.79	0.81	0.63
*Hypoponera ergatandria*	N	11.0	M	–	15.0	M	–	4	0.70	0.75	0.25
*Hypoponera punctatissima*	N	10.0	M	–	16.0	M	–	6	0.72	0.78	0.25
*Lasius neglectus*	Y	36.0	H	TP	36.0	H	TP	0	0.70	0.70	0.75
*Linepithema humile*	Y	51.0	VH	TP	63.0	VH	TP	12	0.87	0.88	0.75
*Monomorium carbonarium*	N	13.5	M	–	13.5	M	–	0	0.74	0.80	0.25
*Monomorium floricola*	Y	11.0	M	–	15.0	M	–	4	0.85	0.87	0.75
*Monomorium pharaonis*	Y	15.0	M	–	15.0	M	–	0	0.78	0.84	0.25
*Nylanderia jaegerskioeldi*	N	15.0	M	–	21.0	H	FP	6	0.71	0.77	0.25
*Nylanderia vividula*	N	13.0	M	–	17.0	H	FP	4	0.73	0.76	0.50
*Paratrechina longicornis*	Y	17.0	H	TP	29.0	H	TP	12	0.77	0.81	0.50
*Pheidole indica*	Y	24.0	H	TP	30.0	H	TP	6	0.79	0.82	0.54
*Pheidole megacephala*	Y	28.0	H	TP	40.0	VH	TP	12	0.71	0.77	0.25
*Plagiolepis alluaudi*	N	25.5	H	FP	31.5	H	FP	6	0.67	0.72	0.25
*Solenopsis geminata*	Y	40.0	VH	TP	46.0	VH	TP	6	0.62	0.67	0.25
*Solenopsis invicta*	Y	45.0	VH	TP	57.0	VH	TP	12	0.91	0.90	1.00
*Strumigenys membranifera*	N	12.0	M	–	12.0	M	–	0	0.76	0.81	0.38
*Tapinoma melanocephalum*	Y	26.0	H	TP	32.0	H	TP	6	0.76	0.79	0.50
*Technomyrmex pallipes*	N	16.0	M	–	16.0	M	–	0	0.71	0.77	0.25
*Tetramorium bicarinatum*	Y	19.0	H	TP	25.0	H	TP	6	0.74	0.80	0.25
*Tetramorium immigrans*	N	28.0	H	FP	28.0	H	FP	0	0.75	0.82	0.25
*Tetramorium lanuginosum*	N	13.0	M	–	13.0	M	–	0	0.75	0.81	0.25
*Trichomyrmex destructor*	Y	20.0	H	TP	24.0	H	TP	4	0.67	0.72	0.29
*Wasmannia auropunctata*	Y	38.5	H	TP	50.5	VH	TP	12	0.73	0.74	0.67

## Data Availability

The data supporting the conclusions of this article are available in the Supplementary Materials.

## Supplementary Materials

The following supporting information can be downloaded at: https://www.mdpi.com/article/10.3390/insects15110875/s1, Table S1: Screenings of established and horizon non-native ant species following the terrestrial species invasiveness screening kit (TAS-ISK).
